# Osteoporotic Fracture Guidelines and Medical Education Related to the Clinical Practices: A Nationwide Survey in China

**DOI:** 10.1111/os.12476

**Published:** 2019-07-19

**Authors:** Jin Lu, Zhong Ren, Xun Liu, You‐jia Xu, Qiang Liu

**Affiliations:** ^1^ Medical Affairs, Osteoporosis Franchise Sandoz China Tianjin China; ^2^ Medical Affairs, Osteoporosis Franchise Sandoz China Chengdu China; ^3^ Medical Affairs, Osteoporosis Franchise Sandoz China Shanghai China; ^4^ Orthopaedic Department, The 2^nd^ Affiliate Hospital of Suzhou University Suzhou China; ^5^ Orthopaedic Department, Shanxi Da Yi Hospital Taiyuan China

**Keywords:** Chinese osteoporotic fracture guidelines, Medical education, Osteoporotic fracture, Questionnaire survey

## Abstract

**Objectives:**

To investigate the knowledge and practices of Chinese doctors in the management of osteoporotic fractures after the Chinese osteoporotic fracture guidelines update and aseries of medical education in 2017.

**Methods:**

This was a cross‐sectional survey of doctors in 71 cities across Mainland China. Based on the 2017 Chinese guidelines for the diagnosis and treatment of osteoporotic fractures, a questionnaire was designed and pre‐tested for reliability and validity. Doctors were surveyed with the questionnaire after scientific meetings during February 2017 to January 2018 through WeChat or conference digital platforms or in paper form. Descriptive statistics was used to analyze the responses to the questionnaire.

**Results:**

Overall, 314 valid questionnaires were confirmed. Regarding diagnosis, 77% agreed that osteoporosis could be diagnosed once an osteoporotic fracture occurred; 83% believed that the bone mineral density criteria for osteoporosis diagnosis would be T ≤ −2.5 SD. For treatment, almost all (99.7%) agreed with anti‐osteoporosis treatment being one of the basic principles of osteoporotic fracture treatment; 71.6% considered bisphosphonates as the most commonly used anti‐osteoporosis drug; 97% believed that patients who have used anti‐osteoporosis drugs should reassess osteoporosis after osteoporotic fractures instead of discontinue; 95% thought that the patients who did not use anti‐osteoporosis medications before osteoporotic fracture should be treated with anti‐osteoporosis drugs after fracture treatment as early as possible; 89% agreed that the standard use of bisphosphonates after osteoporotic fracture would not affect bone healing adversely; 59% believed the course of bisphosphonates treatment for osteoporosis would be 3–5 years, and 27% considered it to be 1–3 years. The patient follow‐up rate was poor: 46% selected follow‐up rate <30%; only 20% selected follow‐up rate >50%. Only 31% of the hospitals had long‐term management programs for osteoporotic fractures.

**Conclusions:**

Doctors generally adhered to the updated Chinese guidelines for osteoporotic fractures; frequent participation in medical education can help doctors to increase their awareness of osteoporosis as well as their acceptance and practice of the guidelines.

## Introduction

Osteoporosis is one of the most common skeletal diseases. It can occur at any age but is more common in postmenopausal women and older men^1^. Severe osteoporosis will eventually lead to osteoporotic fractures. Approximately 40%–50% of females and 13%–22% of males sustain at least one osteoporotic fracture over the course of their entire life^2^. Among the Chinese population, it is estimated that more than one‐third of the women and approximately one‐tenth of the men aged 50 years are expected to sustain a major osteoporotic fracture in their remaining lifetimes^3^. Osteoporotic fractures may happen at various sites of the body, and fracture at some site, such as hip fracture, is associated with high mortality^4^. In addition, the economic burden on patients and the healthcare system resulting from osteoporotic fractures is heavy^5^. Osteoporotic fractures are also significantly associated with a higher risk of subsequent fracture and excess mortality^6^, ^7^. Therefore, prevention and treatment of osteoporotic fractures are particularly important.

To standardize the diagnosis and treatment of osteoporotic fractures, guidelines on osteoporosis and osteoporotic fractures have been developed in various countries; the Chinese osteoporotic fracture guidelines was first released in 2008 and was updated in 2017. However, the prevention and treatment of osteoporosis and osteoporotic fractures have not improved since the release of the guidelines^8^, ^9^, ^10^. In a retrospective patient survey of Chinese women aged over 50 with fragility hip and vertebral fractures, osteoporosis was diagnosed in 56.8% of the patients, and 42.0% had never been assessed for bone mineral density. After the index fracture, 69.6% of the patients received supplements and/or anti‐osteoporotic medications, among which only 39.6% received calcium with or without vitamin D supplementation^8^. The reason for the low rates of diagnosis and treatment for osteoporosis after fracture may be various. The lack of awareness and knowledge of osteoporosis or osteoporotic fractures, which not only exists among the general population but also exists among medical staff^11^, ^12^, ^13^, is an important reason.

Furthermore, multiple pathogenic factors are related to osteoporosis^1^. In addition to the well‐known factors such as gender, age, and calcium and vitamin D deficiency, diseases such as diabetes, rheumatoid arthritis, and gout are associated with osteoporotic fractures^14^, ^15^, ^16^. Doctors in endocrinology departments are usually not aware of the necessity of anti‐osteoporotic treatment for diabetic patients with a high risk of fragility fracture. Therefore, it is necessary to strengthen the education on osteoporosis and osteoporotic fractures for medical staff of the related specialties besides those specializing in osteoporosis or osteology.

To investigate the treatment given to osteoporotic fracture patients by orthopaedists at major hospitals in China, a nationwide survey among Chinese senior orthopaedists was conducted in 2014, and the results were published in 2016^17^. As shown in that survey, there were quite a few orthopaedists lacking knowledge of treatment and management of osteoporotic fractures. Physicians with longer working experience tended to have better treatment knowledge and to manage osteoporotic conditions according to clinical practice guidelines. Following the Chinese osteoporotic fracture guidelines being updated and a series of medical training sessions and meetings having been held, we initiated a survey to investigate the current knowledge and practices of Chinese doctors from multiple specialties on management of osteoporotic fractures, and tried to find any factors that impact on the knowledge and clinical practice of the doctors.

## Material and Methods

### *Design and Setting*

This was a cross‐sectional study conducted in Mainland China. Based on the 2017 Chinese guidelines for the diagnosis and treatment of osteoporotic fractures^18^, a questionnaire containing 22 closed questions and an open question was developed covering the diagnosis and management of osteoporosis and osteoporotic fractures (Table 1). The 22 closed questions were categorized into several domains; the uncategorized open question (Q 22) asked which city the respondent worked in. A pre‐test was taken to investigate the reliability and validity of the questionnaire before the survey began. The pre‐test was conducted by 55 doctors from different cities. Each doctor was asked to respond to the questionnaire twice with an interval of 2–4 weeks to verify the consistency. Thirty‐four valid questionnaires were finally collected. The Cronbach's α measure was used to test internal consistency of the questionnaire items and good internal consistency was observed (Cronbach's α > 0.7). The Kaiser–Meyer–Olkin measure of sampling adequacy and Bartlett's test of sphericity showed that the results were suitable for factor analysis.

**Table 1:** Survey questionnaire

Question category	Question number	Question content	Question options
Osteoporosis diagnosis	Q1	Do you agree that osteoporosis can be diagnosed clinically by the occurrence of fragility fractures?	A. AgreeB. DisagreeC. Not sure
	Q2	The bone mineral density criterion for osteoporosis diagnosis is:	A. T ≥ −1.0 SD B. ‐2.5 SD < T < ‐1.0 SD C. T ≤ −2.5 SD
	Q4	The specific bone turnover biochemical markers recommended by the IOF are:	A. P1NP, S⁃CTXB. Bone alkaline phosphatase, osteocalcinC. Vitamin D, calcium phosphateD. Thyroid hormone, parathyroid hormone
Characteristics and treatment principles of osteoporotic fractures	Q3	Which descriptions of the characteristics of osteoporotic fracture are correct in your opinion? (Choose one or more)	A. Rapid bone loss will occur when a patient stays in bed after fracture, and will aggravate osteoporosis;B. Abnormal bone reconstruction, slow healing process, long recovery time, delayed fracture healing or even non‐healing;C. Significantly increased risk of re‐fracture at the same site or other sites;D. Low bone mass in the fracture site, poor bone quality, and most are comminuted fractures, which is difficult to achieve reduction;E. Poor stability of internal fixation treatment; internal fixation and implants are easy to loosen and escape, and the bone graft is easily absorbed;F. More common in the elderly who are with poor general condition and with other organ diseases or systemic diseases. Complications may occur during treatment and increase the complexity of treatment.
	Q5	The basic principles of treatment of osteoporotic fractures are: (Choose one or more)	A. Reduction B. Fixation C. Functional exercise D. Anti‐osteoporosis treatment
	Q6	The common sites of osteoporotic fracture are: (Choose one or more)	A. Spine B. Hip C. Distal radius D. Proximal humerus
Drug treatment	Q7	The dosages of calcium and vitamin D you usually give to patients in the early stage after osteoporotic fracture are:	A. Ca 400 mg/day, VitD 200 IU/dB. Ca 600 mg/day, VitD 400 IU/dC. Ca 1000 mg/day, VitD 800 IU/dD. Ca 1200 mg/day, VitD 800 IU/dE. Ca >1200 mg/day, VitD 1200 IU/d
	Q8	Which type of anti‐osteoporosis drug is the most prescribed in your clinical practice? (Choose one)	A. Bisphosphonates B. Selective estrogen receptor modulators, estrogens C. Calcitonin D. Anabolic agents E. Active vitamin D F. Chinese patent medicine
	Q9	For patients who have used anti‐osteoporosis drug, should the anti‐osteoporosis drug be discontinued after osteoporotic fracture?	A. Yes B. Reassessing osteoporosis instead of blindly discontinuing the drug C. Unclear
	Q10	Which is the optimal treatment for osteoporotic fracture patients who did not use anti‐osteoporosis drugs before osteoporotic fracture?	A. Use anti‐osteoporosis agents as soon as fracture treatment completed when the patient's general condition is stableB. Start anti‐osteoporosis medication after fracture healingC. Only basic supplement therapy with calcium and vitamin D D. Only use short‐term calcitonin to suppress acute pain
Drug selection	Q11	Do you agree that standardized use of bisphosphonates after osteoporotic fractures would not adversely affect fracture healing?	A. Agree B. Disagree C. Not sure
	Q12	Under what circumstances will you choose anabolic drugs to treat osteoporotic fractures?	A. For patients with osteoporotic fracture treated with anti‐bone resorption drugs for many yearsB. For postmenopausal women who have osteoporotic vertebral fractures or hip fractures for many timesC. For patients with multiple osteoporotic fracturesD. Not to be used
Medication time	Q13	Which of the following statements do you agree about the effects of bisphosphonates on fracture healing and internal plants? (Choose one or more)	A. Use of bisphosphonates after osteoporotic fracture may lead to increased osteophytes, increased mineralization, and no delayed fracture healing.B. Use of bisphosphonates after internal fixation can inhibit further loss of bone mass, improve stability of internal fixation, and reduce incidence of internal fixation displacement.C. Use of bisphosphonates after artificial joint replacement for osteoporotic hip fracture can increase hip bone mass, reduce bone loss around the prosthesis, and reduce incidence of prosthesis loosening.
Duration of treatment	Q14	Which do you think is the reasonable duration of bisphosphonates treatment for patients with osteoporosis?	A. <3 monthsB. 3–12 months (including 12 months)C. 1–3 years (including 3 years)D. 3–5 yearsE. Not sure
Patient management	Q15	For osteoporosis patients after fracture healing, which department do you usually advise them to visit to continue anti‐osteoporosis treatment?	A. OrthopaedicsB. EndocrinologyC. RehabilitationD. GeriatricsE. OsteoporosisF. RheumatologyG. Chinese Medicine
	Q16	What is the revisit and follow‐up rate in patients with osteoporotic fracture in your clinical practice?	A. <30% B. 30%–50% C. >50%
	Q17	Does your hospital have any system, personnel or program for long‐term management of osteoporotic fractures?	A. Yes B. No C. Under arrangement
Feedback of respondents	Q18	Are you satisfied with the content and discussion of this meeting? Please provide your valuable comments and suggestions.	A. Satisfied B. Fair C. Unsatisfied
	Q19	What is your expertise?	A. SpineB. JointC. TraumaD. General orthopaedics and othersE. EndocrineF. RheumatismH. GeriatricsI. OsteoporosisJ. General internal medicine and others
	Q20	What is your job title?	A. Chief physician B. Deputy chief physician C. Attending physician D. Resident physician
	Q21	What is the grade of your hospital?	A. Tertiary hospital B. Secondary hospital C. Primary hospital
	Q23	How many times have you attended osteoporosis‐related training or meetings in the past year?	A. <3 timesB. 3–7 timesC. >7 times

### *Participants*

Doctors who were from departments related to bone or aged patients, such as orthopaedics, trauma, endocrine, rheumatism, geriatrics or osteoporosis, and who attended medical scientific meetings were eligible to participate in this survey (Table 2). There was no limit for sex, age, work experience, or job title.

**Table 2:** Characteristics of respondents

Variable	Category	Number of respondents	%
Grade of the hospital working in	Tertiary hospital	260	83%
	Secondary hospital	53	17%
	Primary hospital	1	0%
Job title	Chief physician	63	20%
	Deputy chief physician	92	29%
	Attending physician	110	35%
	Resident physician	49	16%
Specialty	Spine	101	32%
	Joint	35	11%
	Trauma	36	12%
	General orthopaedics and others	10	3%
	Endocrine	59	19%
	Rheumatism	44	14%
	Geriatrics	15	5%
	Osteoporosis	13	4%
	General internal medicine and others	1	0%
Frequency of attending medical education*	<3 times	161	51%
	3–7 times	122	39%
	>7 times	30	10%

* Total 313 valid responses.

### *Data Collection*

Doctors were surveyed with the questionnaire during or after non‐promotional medical scientific meetings held by Medical Affairs Sandoz China during February 2017 to January 2018 through WeChat or conference digital platforms or in paper form. Completed questionnaires were excluded if they had >10% missing data or essential information was missing.

### *Statistical Analysis*

Descriptive analysis of the questionnaire data was performed using SPSS 20.0 software (SPSS, Chicago, IL, USA). Pearson's χ^2^‐test and percentages of the total were used for analysis. Additional sub‐analysis by cohort based on the respondents’ job title and frequency of attending medical education was also performed.

## Results

A total of 316 questionnaires were completed by participants from 24 provinces, including 71 cities across Mainland China. Of these, 314 (99%) responses (218 from WeChat, 72 in paper form, and 24 from conference digital platforms) were used for the analysis; another 2 responses were excluded due to the exclusion criteria.

### *Characteristics of Respondents*

The characteristics of the respondents are shown in Table 2. Among the 314 respondents, 63 (20%) were chief physicians, 92 (29%) were deputy chief physicians, 110 (35%) were attending physicians, and 49 (16%) were resident physicians. The majority specialized in spine (101, 32%), endocrine (59, 19%), rheumatism (44, 14%), joints (35, 11%), or osteoporosis (13, 4%). Most (260, 83%) of them were from tertiary hospitals. Nearly half (161, 51%) of the doctors had attended osteoporosis‐related training or meetings in the past year fewer than three times, and 30 (10%) respondents had attended training or meetings more than seven times.

### *Diagnosis of Osteoporosis*

**Figure 1:**
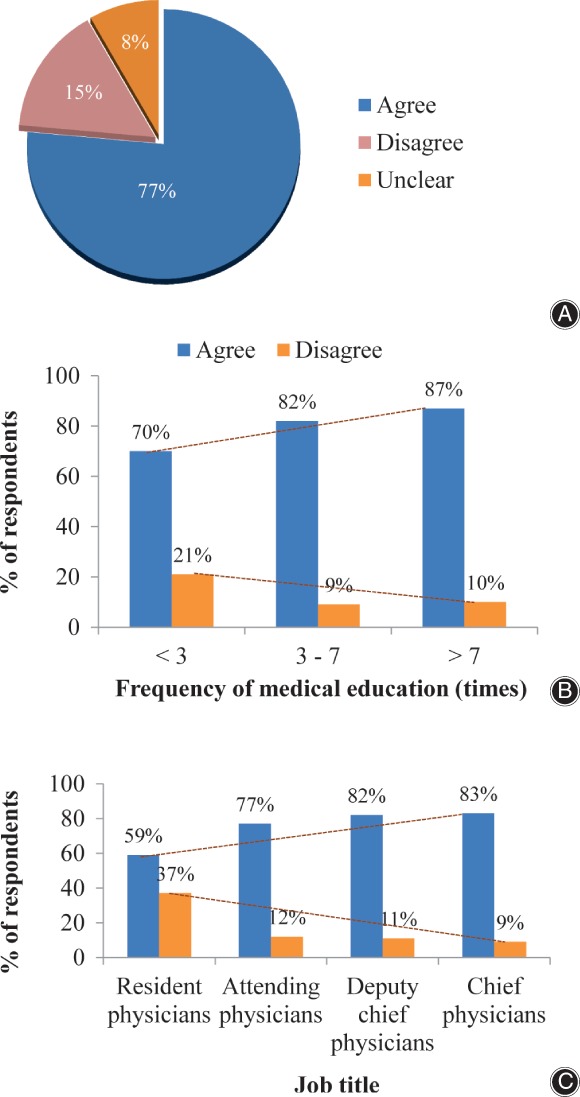
Responses to Q1 (Do you agree that osteoporosis can be diagnosed clinically by the occurrence of fragility fractures?). (A) Overall analysis; (B) analysis by frequency of medical education; (C) analysis by job title.

In response to the question “Do you agree that osteoporosis can be diagnosed clinically by the occurrence of fragility fractures?” most (77%) of the respondents chose “agree”; 15% disagreed and 8% were not sure. Sub‐group analysis showed that the percentage of respondents who chose “agree” increased with the frequency of attending medical education: 70%, 82%, and 87% in groups of <3 times, 3–7 times, and >7 times respectively. When analyzed according to job title, the percentage of respondents answering yes was 59%, 77%, 82% and 83% among resident physician, attending physician, deputy chief physician and chief physician groups, respectively (Fig. 1).

Eighty‐three percent of the respondents believed that the bone mineral density criteria for osteoporosis diagnosis would be T ≤ −2.5 standard deviation (SD), and 15% believed it should be −2.5 SD < T < −1.0 SD. Seventy‐five percent thought procollagen type I N‐terminal propeptide (P1NP) and collagen type I crosslinked C‐telopeptide (S⁃CTX) should be the specific bone turnover biochemical markers recommended by the International Osteoporosis Foundation.

### *Characteristics and Treatment Principles of Osteoporotic Fractures*

With regard to the characteristics of osteoporotic fractures (Q3), the majority of respondents had good awareness; the selection percentage of option A, B, C, D, E, and F was 96%, 93%, 93%, 89%, 90%, and 91%, respectively. Almost all (99.7%) agreed that anti‐osteoporosis treatment should be one of the basic principles of osteoporotic fracture treatment; the percentage of respondents who picked “reduction,” “fixation,” and “functional exercise” was 87%, 88%, and 90% respectively. The rates of respondents regarding the spine and hip as the most common sites of osteoporotic fractures were 97% and 92%, respectively, while the rates of those choosing distal radius and proximal humerus were 83% and 67%, respectively.

### *Drug Treatment*

**Figure 2:**
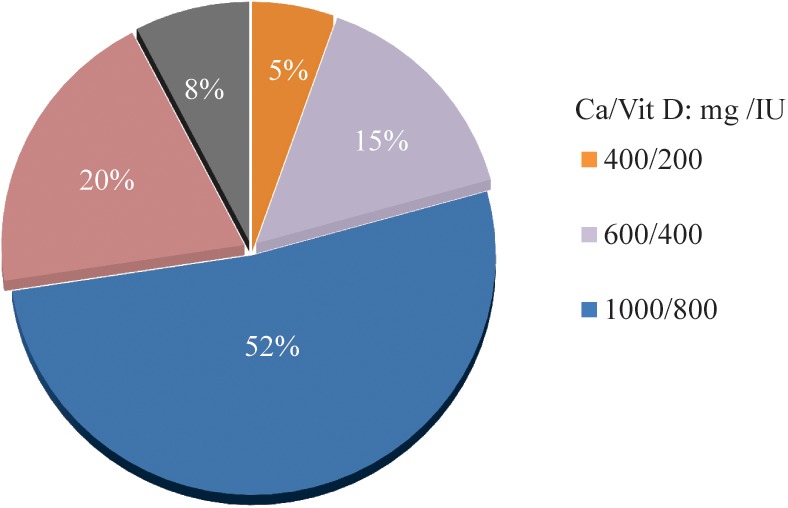
The most prescribed daily dosages of calcium and vitamin D.

Among the respondents, 52% recommended that the daily dosage of calcium and vitamin D early after osteoporotic fractures should be 1000 mg and 800 IU, respectively; 20% thought the daily dosage should be 1200 mg and 800 IU, respectively (Fig. 2). There was no significant difference in the distribution of options among groups, and it did not correlate with the frequency of attending medical education or the job title of respondents.

**Figure 3:**
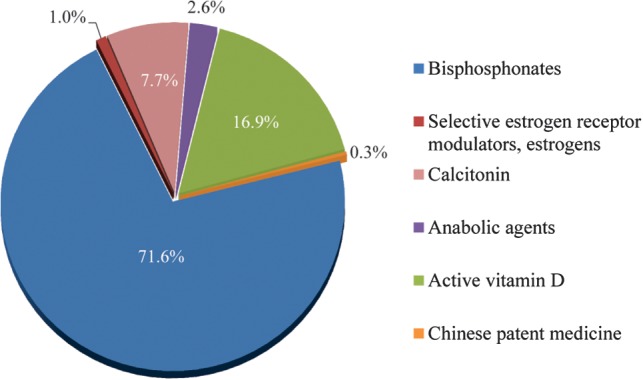
The most prescribed anti‐osteoporosis drugs.

**Figure 4:**
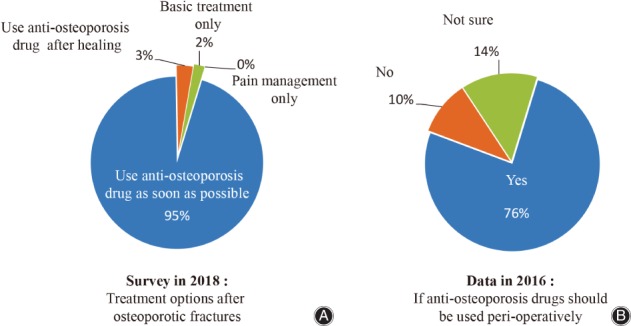
The knowledge on timing of anti‐osteoporosis medication.

Among the respondents, 71.6% considered bisphosphonates as the most commonly used anti‐osteoporosis drug; active vitamin D was the second most frequently used drug (Fig. 3). Ninety‐seven percent believed that patients who have used anti‐osteoporosis drugs should reassess osteoporosis after osteoporotic fracture instead of discontinuing; 95% agreed that the patients who did not use anti‐osteoporosis medications before osteoporotic fracture should be treated with anti‐osteoporosis drugs as soon as fracture treatment was completed (Fig. 4A).

### *Drug Selection, Medication Time, and Duration*

Most (89%) of the respondents agreed that standardized use of bisphosphonates after osteoporotic fractures would not adversely affect fracture healing. For the question on the application scenarios of anabolic drugs, 63% would use this kind of drug for patients with osteoporotic fractures treated with anti‐bone resorption drugs for many years, and 26% would use it many times for postmenopausal women who have osteoporotic vertebral fractures or hip fractures.

**Figure 5:**
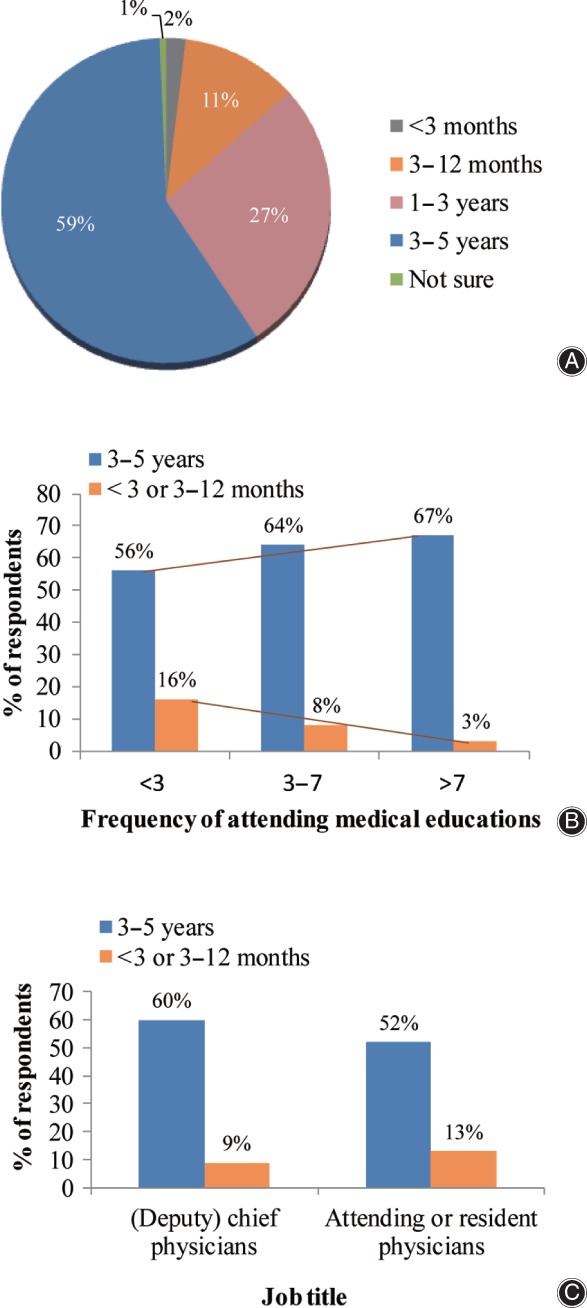
Recommended duration of bisphosphonates treatment. (A) Overall analysis; (B) analysis by frequency of attending medical education; and (C) analysis by job title.

Regarding the effects of bisphosphonates on fracture healing and internal implants (Q13), most of the respondents agreed on their positive effects (81%, 90%, and 86% for options A, B, and C, respectively). Regarding the duration of bisphosphonates treatment, 59% of the respondents suggested the reasonable duration for osteoporosis treatment should be 3–5 years, and the option of 1–3 years was chosen by 27% of the respondents. With the frequency of medical education increased, more respondents agreed that the duration of bisphosphonates treatment should be 3–5 years, as recommended by the guidelines, and fewer respondents would prescribe for less than 1 year. Meanwhile, more respondents in the chief physician and deputy chief physician group agreed on 3–5 years compared with the group of attending physicians and resident physicians (Fig. 5).

### *Patient Management*

**Figure 6:**
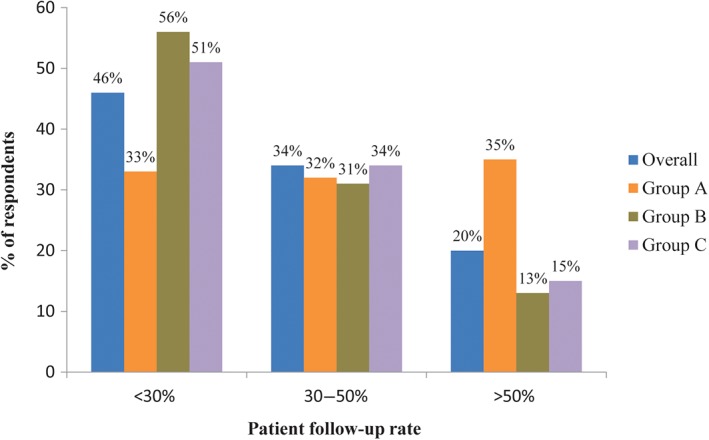
Follow‐up rate of patients with osteoporotic fracture. Group A: doctors from hospitals with long‐term management program; Group B: doctors from hospitals without long‐term management program; Group C: doctors from hospitals with long‐term management programs at planning or preparatory stage.

For subsequent therapy after fracture healing, 37% and 38% of the respondents suggested the patients visit orthopaedics and osteoporosis departments, respectively, and 16% recommended visiting an endocrinology department. Thirty‐one percent of the hospitals the respondents were working in had long‐term management programs for osteoporotic fractures, while 37% had no related program and the other 32% had programs at planning or preparatory stage. With respect to patient follow‐up, nearly half (46%) of the respondents had a follow‐up rate of <30%, and 20% had a rate of >50%. A larger percentage of respondents in the group attending medical education >7 times had a patient follow‐up rate >50% compared with the groups attending medical education 3–7 times and <3 times (37%, 15% and 20%, respectively). The chief physician group had the largest percentage (27%) of patient follow‐up rate >50%. The patient follow‐up rate was higher in hospitals having long‐term management programs for osteoporotic fractures than in hospitals with no related program or with programs at planning or preparatory stage (Fig. 6).

## Discussion

Osteoporosis‐related fractures are a worldwide public health problem. With the increase in the elderly population, more and more osteoporosis and osteoporotic fracture patients will emerge. The level of knowledge of medical staff has important impacts on disease management. Prior studies in China have shown that although most orthopaedic doctors are aware of the necessity of anti‐osteoporosis therapy for osteoporotic fractures, more effort should be made to improve their knowledge on disease screening, diagnosis, and treatment^19^, ^20^. In this survey we investigated the knowledge of doctors in multiple related specialties following updates of the guidelines and increased medical education.

With regard to the dosage of calcium and vitamin D, approximately half of the respondents would prescribe 1000 mg/day for calcium and 800 IU/day for vitamin D, as recommended in the updated guidelines, compared with 32% choosing 1000 mg/day for calcium and 49.6% choosing 800 IU/day for vitamin D in the 2016 survey. There was no obvious difference in the distribution of options between overall and sub‐group analysis, and it was not related to the frequency of attending medical education or job title. This could be explained by several possible reasons, including: it was not emphasized enough on this topic during medical training or meetings; the accustomed prescription had been able to meet the treatment needs for patients; and there may also be geographical differences.

**Figure 7:**
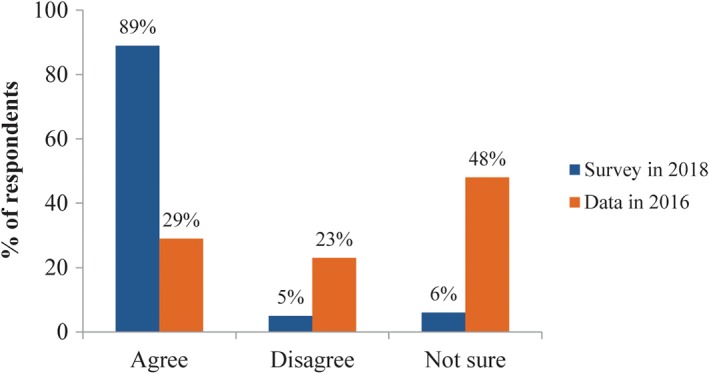
Responses to Q11 (Do you agree that standardized use of bisphosphonates after osteoporotic fractures would not adversely affect fracture healing?), comparing with the data published in 2016.

**Figure 8:**
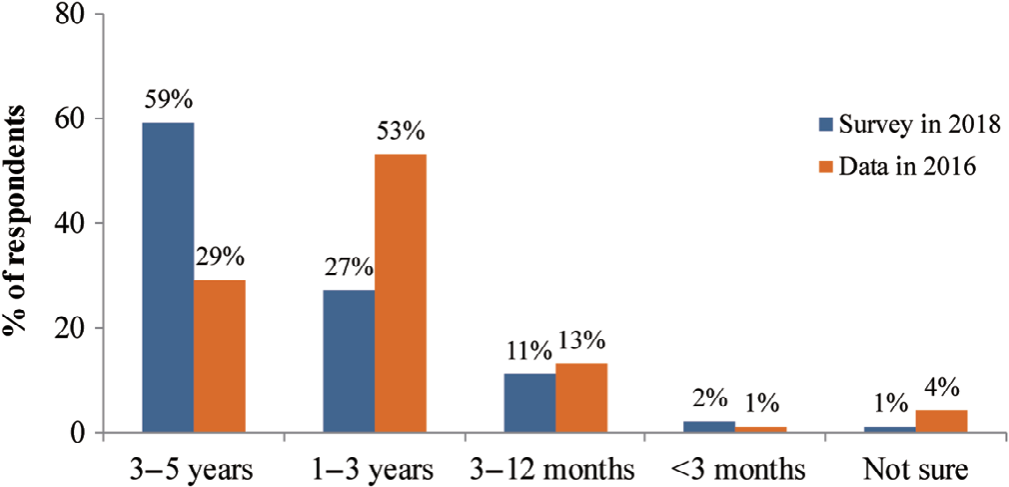
The knowledge on duration of bisphosphonates treatment, comparing with the data published in 2016.

Compared with the survey published in 2016, more respondents agreed that standardized bisphosphonates therapy has no adverse effect on fracture healing after osteoporotic fractures (89% *vs* 29.3%) (Fig. 7). The majority (95%) of respondents in this survey agreed to use anti‐osteoporosis drugs as soon as possible after fracture treatment when the patients’ general condition was stable, whereas 76% in the 2016 survey agreed to use anti‐osteoporosis drugs during the perioperative period (Fig. 4). More respondents in this survey suggested 3–5 years, as the guidelines recommended, would be the reasonable duration for bisphosphonates treatment compared to 2016 (59% *vs* 29%) (Fig. 8). Sub‐analysis showed the percentage of respondents who agreed that the recommended duration of 3–5 years increased with the frequency of medical education. According to the respondents’ job title, the proportion of bisphosphonates prescribed for 3–5 years by residents and attending physicians was less than that by chief physicians and deputy chief physicians (52% *vs* 60%). At the same time, the attending physician group tends to have the largest proportion of bisphosphonates prescriptions for 1–3 years, while the prescription by resident physicians has the largest proportion within 1 year. The reasons may be related to the differences in the degree of doctors’ knowledge, the degree of patients’ conviction, and prescribing authority.

Anti‐osteoporosis treatment is one of the basic principles of osteoporotic fracture treatment; however, patient compliance has been a problem^21^, ^22^. For patients receiving treatment for osteoporotic fractures, those without subsequent visits or with loss of follow‐up may have poor compliance. Our study showed that the patient follow‐up rate was influenced by the frequency of attending medical education; it may be because the doctors who attended medical training more frequently had more awareness of patient adherence. The influence of the doctors’ job title on the patient follow‐up rate may be more due to the patient's trust in high‐ranking physicians. It is worth mentioning that the patient follow‐up rates of respondents from hospitals with long‐term management programs for osteoporotic fractures were much higher, suggesting that long‐term programs for osteoporotic fracture management in hospitals are critical for follow‐up visits and patient compliance. For example, a fracture liaison service, such as that launched by the International Osteoporosis Foundation in 2012, can improve patient adherence and was proved to be an effective model of care for patients with osteoporotic fractures^23^.

Through comparison of the results of the survey published in 2016, we found that the awareness and the level of diagnosis and treatment of osteoporosis of doctors have been improved. The diagnosis and treatment of osteoporosis and long‐term management of osteoporotic fractures should be based on doctors’ knowledge and patient compliance. We believe that such results are meaningful; they are also helpful for government and enterprises to develop policies and strategies on osteoporosis management. Meanwhile, we call for strengthening medical education for both medical staff and the general population to prevent osteoporosis and reduce fracture incidence.

This questionnaire survey was conducted mainly through WeChat and online methods, which improved the rate of valid questionnaires and avoided interference caused by human factors in the face‐to‐face questionnaire form. However, as this was a cross‐sectional survey with treatment data retrieved from doctors’ subjective clinical concepts instead of clinical records, personal biases may exist and affect the results.

### *Conclusions*

The results of this survey demonstrated that doctors knowledge about osteoporosis and osteoporotic fractures improved in relation to diagnosis, bisphosphonates and bone healing, timing of medication and long‐term treatment, and generally adhered to the updated Chinese guidelines for osteoporotic fractures. Frequent medical education can help doctors to enhance the awareness of osteoporosis and osteoporotic fractures as well as the acceptance and practice of guidelines. Long‐term management programs for osteoporotic fractures in hospitals may be useful for improving patient compliance and optimizing disease management.
